# Phylogeny and Immunoreactivity of Norovirus GII.P16-GII.2, Japan, Winter 2016–17

**DOI:** 10.3201/eid2401.170284

**Published:** 2018-01

**Authors:** Koo Nagasawa, Yuki Matsushima, Takumi Motoya, Fuminori Mizukoshi, Yo Ueki, Naomi Sakon, Koichi Murakami, Tomomi Shimizu, Nobuhiko Okabe, Noriko Nagata, Komei Shirabe, Hiroto Shinomiya, Wataru Suzuki, Makoto Kuroda, Tsuyoshi Sekizuka, Akihide Ryo, Kiyotaka Fujita, Kazunori Oishi, Kazuhiko Katayama, Hirokazu Kimura

**Affiliations:** National Institute of Infectious Diseases,Tokyo, Japan (K. Nagasawa, K. Murakami, M. Kuroda, T. Sekizuka, K. Oishi, H. Kimura);; Kawasaki City Institute for Public Health, Kanagawa, Japan (Y. Matsushima, T. Shimizu, N. Okabe);; Ibaraki Prefectural Institute of Public Health, Ibaraki, Japan (T. Motoya, N. Nagata);; Tochigi Prefectural Institute of Public Health and Environmental Science, Tochigi, Japan (F. Mizukoshi);; Miyagi Prefectural Institute of Public Health and Environment, Miyagi, Japan (Y. Ueki);; Osaka Prefectural Institute of Public Health, Osaka, Japan (N. Sakon);; Yamaguchi Prefectural Institute of Public Health and Environment, Yamaguchi, Japan (K. Shirabe);; Ehime Prefectural Institute of Public Health and Environmental Science, Ehime, Japan (H. Shinomiya);; Eiken Chemical Co. Ltd., Tochigi (W. Suzuki);; Yokohama City University Graduate School of Medicine, Kanagawa (A. Ryo, H. Kimura);; Gumma Paz University, Gunma, Japan (K. Fujita);; Kitasato University, Minato-ku, Tokyo (K. Katayama)

**Keywords:** norovirus, capsid, RNA-dependent RNA polymerase, phylogeny, immunoreactivity, winter, Japan, viruses

## Abstract

During the 2016–17 winter season in Japan, human norovirus GII.P16-GII.2 strains (2016 strains) caused large outbreaks of acute gastroenteritis. Phylogenetic analyses suggested that the 2016 strains derived from the GII.2 strains detected during 2010–12. Immunochromatography between 2016 strains and the pre-2016 GII.2 strains showed similar reactivity.

Norovirus is a major cause of acute gastroenteritis in humans and is genetically classified into 7 genogroups (GI–GVII). Among them, norovirus GI, GII, and GIV infect humans, and human norovirus (HuNoV) has many genotypes (*1*). GII viruses are common and have 22 confirmed genotypes (*1*).

In Infectious Diseases Weekly Report (https://www.niid.go.jp/niid/en/idwr-e.html), the Japanese national surveillance system reports the number of patients with acute gastroenteritis and their pathogenic agents, who were examined at clinics or hospitals of the sentinel surveillance medical institutes (≈3,000 institutions). This report indicated that HuNoV GII.2 was the third or fourth most prevalent genotype during the past 5 seasons (2011–2016), but became the predominant strain during the 2016–17 season. Of note the total number of children with acute gastroenteritis in 2016–17 was the second largest over the past 11 epidemiologic seasons (*2**,**3*).

On the basis of these observations, we used the full length of the RNA-dependent RNA polymerase (RdRp) region and capsid (VP1) gene to study the phylogeny of HuNoV strains detected during 2016–17 winter season (2016 strains). We also determined the immunoreactivities of the variant strains by various immunochromatography (IC) kits and the bioluminescent enzyme immunoassay (BLEIA).

## The Study

In this study, we analyzed 26 GII.2 strains detected during October 2016–January 2017. Of those, we analyzed 19 strains for their phylogeny, and examined 7 strains by IC and BLEIA (*4*). We collected samples from the patients (children and adults) with acute gastroenteritis (mean ±SD age 9.2 ± 12.0 years). Because we could not collect adequate amounts of fecal specimens in some instances, we could not perform sequence analyses for all of them. Moreover, to compare the sensitivities of IC and BLEIA for the previous and current GII.P16-GII.2 strains, we conducted these tests using 2 fecal specimens containing GII.P16-GII.2 virus detected in 2009. These specimens, stored at −80°C, were obtained from clinics or hospitals, such as the sentinel surveillance medical institutions in Ibaraki Prefecture and Kawasaki City in Japan. We obtained written informed consent from the patients or their guardians for the samples. The study protocols were approved by the National Institute of Infectious Diseases for Public Health Ethics Committees (No. 576). 

We performed viral RNA extraction and reverse transcription PCRs as described (*5*). To analyze the complete RdRp region and VP1 gene, we used primer-walking methods with primers designed by the PrimaClade server (Technical Appendix Table 1) (*6*) and performed sequencing as described (*5*). To determine the genotype of the current virus, we used Norovirus Genotyping Tool version 1.0 (*7*). The accession numbers of the current strains are listed in Technical Appendix Table 2. To construct the phylogenetic tree, we collected complete sequences of the VP1 gene and RdRp region of HuNoV from GenBank (Technical Appendix Table 2). We used 61 reference strains for phylogenic analysis of the VP1 gene and 70 strains for the RdRp region. We selected the best substitution models using the Bayesian information criterion (BIC) method by MEGA 6.0 (http://www.megasoftware.net (*8*). Using sequences from both regions, we inferred phylogenetic trees by the maximum-likelihood method as implemented in MEGA 6.0 (*8*).

To explore the immunoreactivities of 2016 strains, we used 7 fecal specimens from the patients with acute gastroenteritis attributed to the 2016 strains to assess relationships among these strains’ genome copy numbers and the sensitivities of 5 commercial IC kits: ImmunoCatch-Noro (Eiken Chemical, Tokyo, Japan); Quick Chaser-Noro (Mizuho Medy, Tosu-shi, Japan); Quick Navi-Noro 2 (Denka Seiken, Tokyo, Japan); GE test Noro Nissui (Nissui Pharmaceutical, Tokyo, Japan); RIDA QUICK Norovirus (R-Biopharm AG, Darmstadt, Germany). In addition, we evaluated the BLEIA (Eiken Chemical, Tokyo) to compare sensitivities for the IC kits to the 2016 strains. We quantified HuNoV genome numbers by using a real-time PCR (*9*). Furthermore, to test the sensitivity of the IC kits and BLEIA with the GII.2 strains before and during the 2016–2017 winter season, we used 2 fecal specimens collected in 2009–10.

The genotype of HuNoV detected in the 2016–17 season was GII.P16-GII.2 (Figure). The maximum-likelihood tree of the VP1 genes formed many clusters (Figure, panel A). The 2016 strains diverged from common ancestors of the GII.P16-GII.2 cluster detected in 2010–12. The phylogenetic tree of the RdRp coding region also formed many clusters (Figure, panel B). The common ancestors of GII.P16 strains diverged into 2 clusters; one contains strains with capsid genotypes GII.3, GII.4, GII.13, and GII.2 detected in 2010–12, and the other one contains strains with capsid genotypes GII.17 and GII.2 detected in 2009–10 and 2012–15. On the basis of these trees, the 2016 strains could have diverged from GII.2 detected in 2010–12. Finally, RdRp of the 2016 strains diverged from the common ancestors of the 2016 strains and GIIP16-GII.4.

**Figure:**
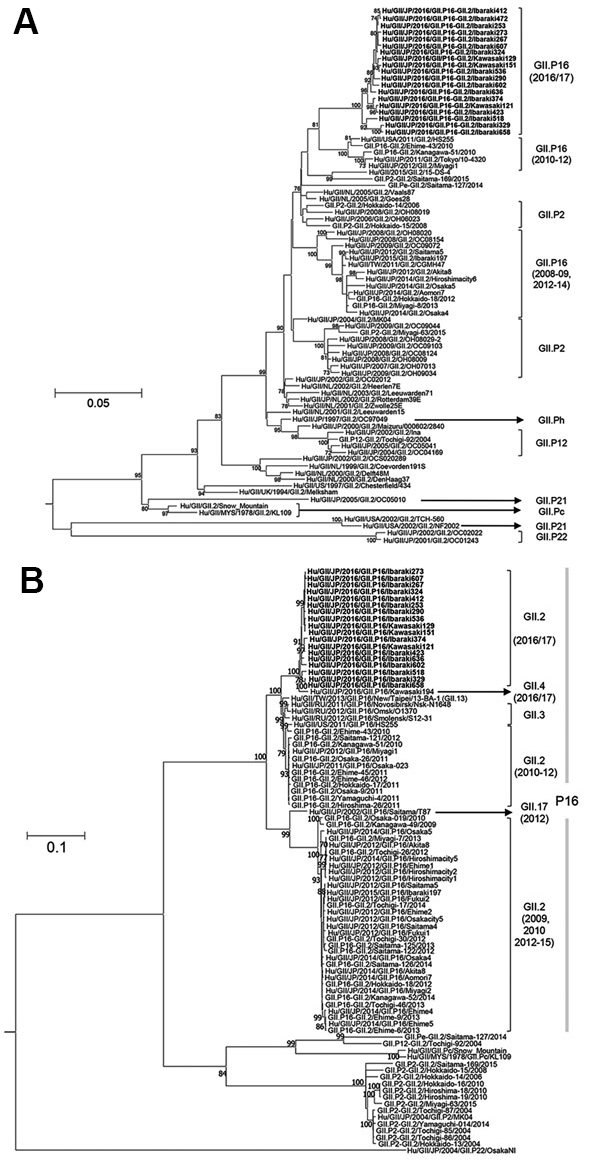
Phylogenetic trees for the A) capsid (VP1) gene and B) RNA-dependent RNA polymerase (RdRp) region in human norovirus GII strains. Trees were constructed by using the maximum-likelihood method. Bold letters denote GII.2v strains. Numbers at branch nodes show bootstrap values with >70% support. Scale bar represents number of nucleotide substitutions per site.

To examine the antigenicity between 2016 strains and pre-2016 GII.2 strains, we assessed the relationship between the quantities of GII.2 genome and the results of the IC kits and BLEIA (Table). The IC kits we used in this study all showed the 2016 strains positive, when ≈10^10^ copies/g of viral genome were in each sample. The reactivities of these kits against lower copy numbers of 2016 strains and pre-2016 GII.2 strains were significantly different. For example, 1 kit (ImmunoCatch-Noro) was >100 times more sensitive than another kit (GE test Noro Nissui) used in this study. BLEIA could detect samples with 2016 strains of genome copy number of ≈2.3×10^6^ copies/g in fecal samples.

**Table:** Sensitivities of 5 immunochromatography kits and the BLEIA for 2016 and pre-2016 GII.P16-GII2 norovirus strains

Sample ID	Virus genome, copies/g	Norovirus immunochromatography kit test results					BLEIA result
		ImmunoCatch-Noro	Quick Chaser-Noro	Quick Navi-Noro 2	GE test Noro Nissui	RIDA QUICK	
Kawasaki181, 2016 strains	9.12 × 10^9^	+	+	+	+	+	+
Kawasaki159, 2016 strains	7.47 × 10^8^	+	+	+	–	+	+
Kawasaki163, 2016 strains	2.30 × 10^7^	+	+	–	–	+	+
Kawasaki173, 2016 strains	1.31 × 10^7^	+	–	–	–	–	+
Kawasaki125, 2016 strains	2.26 × 10^6^	–	–	–	–	–	+
Kawasaki175, 2016 strains	1.82 × 10^6^	–	–	–	–	–	–
Kawasaki177, 2016 strains	3.16 × 10^5^	–	–	–	–	–	–
Ibaraki09–1095, pre-2016 GII.P16-GII.2	8.09 × 10^7^	+	–	–	–	–	+
Ibaraki09–965, pre-2016 GII.P16-GII.2	1.40 × 10^7^	+	–	–	–	–	+

*Kit manufacturers: ImmunoCatch-Noro, Eiken Chemical, Tokyo, Japan; Quick Chaser-Noro, Mizuho Medy, Tosu-shi, Japan; Quick Navi-Noro 2, Denka Seiken, Tokyo, Japan; GE test Noro Nissui, Nissui Pharmaceutical, Tokyo, Japan; RIDA QUICK Norovirus, R-Biopharm AG, Darmstadt, Germany. BLEIA, bioluminescent enzyme immunoassay; ID, identification information; +, positive; –, negative.

## Conclusions

We describe the phylogeny and immunoreactivity of the strains that suddenly emerged in Japan in 2016. As shown in the Figure, the 2016 strains diverged from the HuNoV GII.P16-GII.2 detected in 2010–12, rather than that detected in 2015. Tohma et al. showed that the GII.P16 strains circulating in the 2016–17 winter season carried 4 amino acid mutations (S293T, V332I, K357Q, and T360A) in their polymerase from pre-2016 GII.P16-GII.2 strains (*10*). Among them, all 2016 strains had amino acid mutations (S293T, K357Q, and T360A), and 17 of the 19 strains also had the V332I mutation. Moreover, some reports suggested that the GII.P16-GII.2 strains detected in 2016 were a new recombinant strain, and both the RdRp region and VP1 gene sequences of our strains and the new strains were similar (identity ≈98%–99%, data not shown), although the analyzed sequence lengths were different (*11**,**12*). However, in this study, we could not obtain clear data for the recombinant strains.

Previous reports showed that IC kits were positive for most of the HuNoV GII genotypes in clinical samples (fecal specimens), corresponding to around 10^7^ copies of norovirus genome per gram in the fecal specimen (*13**,**14*). In contrast, another report showed that the reactivity of the pre-2016 GII.2 strains was lower than other genotypes (*15*). We obtained similar data on the sensitivity in each kit between the pre-2016 GII.P16-GII.2 and 2016 strains. However, we saw substantially different sensitivities among these IC kits (maximum 100-fold). Our findings show that the IC kits may be valuable for detecting the GII.P16-GII.2 strains, including pre-2016 GII.2 and 2016 strains on a case-by case basis or as a backup test in the laboratories with a rapid bedside norovirus diagnosis.

The 2016 NoV strains caused outbreaks of acute gastroenteritis in many countries (*2**,**10**–**12*). Additional molecular epidemiologic analyses are needed to track the epidemics and to better understand the viral evolution.

Technical Appendix Primers and reference strains used in study of GII.P16-GII2 norovirus strains, Japan, winter 2016–17.
