# Supplementary material for: Phylogeny and Immunoreactivity of Norovirus GII.P16-GII.2, Japan, Winter 2016–17
Source: Emerg Infect Dis. 2018 Jan;24(1):144–8. doi: 10.3201/eid2401.170284 (PMC5749477; doi:10.3201/eid2401.170284)
Supplement: Technical Appendix — Primers and reference strains used in study of GII.P16-GII2 norovirus strains, Japan, winter 2016–17. [file 17-0284-Techapp-s1.pdf]

Article DOI: https://doi.org/10.3201/eid2401.170284

Phylogeny and Immunoreactivity of Norovirus GII.P16-GII.2, Japan, Winter 2016–17

Technical Appendix

Technical Appendix Table 1. Primers used in this study

Coding region	Primer	Sequence, 5′→3′
RdRp	GII.P16-PCR-1F	CCCAAARCCAATYAGACCAGATGT
	GII.P16-Seq-1F	AGAATGGGRACACACGCAA
	GII.P16-Seq-2F	AYGAGCCTGCCTACCTYGG
	GII.P16-Seq-3F	HCTGCTYTGGGGCTCTGA
	GII.P16-Seq-4F	AAGTCACCAAHCTGTCYCCTGACAT
	GII.P16-Seq-5F	ACTYAAAGAAGGTGGGATGGACT
	G2-SKR (*1*)	CCRCCNGCATRHCCRTTRTACAT
VP1	GII.2-PCR-Seq-1F	AYYTGAGCACGTGGGAGG
	G2-SKF (*1*)	CNTGGGAGGGCGATCGCAA
	G2-SKR (*1*)	CCRCCNGCATRHCCRTTRTACAT
	GII.2-Seq-2F	YAAGTTRGTCTTCGCCGC
	GII.2-Seq-3F	CCAGTGTCYATAGAYCAGATGTAC
	GII.2-Seq-4R	CAGAAGGGGCRAGRTTTGT
	GII.2-PCR-Seq-5R	GAACYRAGCCCATTGCTGA

Technical Appendix Table 2. Norovirus strains used in this study

Strain	GenBank accession no.	Coding region
Pre-2016 strains		
Hu/GII/JP/2011/GII.P16-GII.2/Osaka9	LC209448	RdRp
Hu/GII/JP/2012/GII.P16-GII.2/Miyagi1	LC145787	RdRp, VP1
Hu/GII/JP/2011/GII.P16-GII.2/Yamaguchi4	LC209468	RdRp
Hu/GII/JP/2011/GII.P16-GII.2/Osaka023	LC209453	RdRp
Hu/GII/JP/2011/GII.P16-GII.2/Ehime45	LC209479	RdRp
Hu/GII/JP/2011/GII.P16-GII.2/Hiroshima26	LC209471	RdRp
Hu/GII/JP/2010/GII.P16-GII.2/Kanagawa51	LC209459	RdRp, VP1
Hu/GII/JP/2011/GII.P16-GII.2/Hokkaido17	LC209467	RdRp
Hu/GII/JP/2012/GII.P16-GII.2/Ehime46	LC209478	RdRp
Hu/GII/JP/2011/GII.P16-GII.2/Osaka26	LC209449	RdRp
Hu/GII/JP/2012/GII.P16-GII.2/Saitama121	LC209446	RdRp

Strain	GenBank accession no.	Coding region
Hu/GII/JP/2011/GII.2/Tokyo/10–4320	AB629946	VP1
Hu/GII/JP/2010/GII.P16-GII.2/Ehime43	LC209481	RdRp, VP1
Hu/GII/U.S./2011/GII.P16-GII.2/HS255	KJ407074	RdRp, VP1
Hu/GII/JP/2014/GII.P2-GII.2/Yamaguchi014	LC209469	RdRp
Hu/GII/JP/2004/GII.P2-GII.2/Tochigi87	LC209437	RdRp
Hu/GII/JP/2004/GII.P2-GII.2/Tochigi86	LC209438	RdRp
Hu/GII/JP/2004/GII.P2-GII.2/Tochigi85	LC209436	RdRp
Hu/GII/JP/2004/GII.P2-GII.2/Hokkaido13	LC209464	RdRp
Hu/GII/JP/2004/GII.P2-GII.2/MK04	DQ456824	RdRp, VP1
Hu/GII/JP/2002/GII.2/OC02012	AB279555	VP1
Hu/GII/NL/2002/GII.2/Heerlen7E	AB281086	VP1
Hu/GII/NL/2002/GII.2/Rotterdam39E	AB281087	VP1
Hu/GII/NL/2001/GII.2/Zwolle25E	AB281085	VP1
Hu/GII/NL/2003/GII.2/Leeuwarden71	AB281088	VP1
Hu/GII/JP/2006/GII.P2-GII.2/Hokkaido14	LC209462	RdRp, VP1
Hu/GII/NL/2005/GII.2/Vaals87	AB281090	VP1
Hu/GII/NL/2005/GII.2/Goes28	AB281089	VP1
Hu/GII/JP/2006/GII.2/OH06023	AB662863	VP1
Hu/GII/JP/2008/GII.2/OH08019	AB662867	VP1
Hu/GII/NL/2001/GII.2/Leeuwarden15	AB281084	VP1
Hu/GII/JP/1997/GII.2/OC97049	AB279553	VP1
Hu/GII/JP/2010/GII.P2-GII.2/Hokkaido16	LC209465	RdRp
Hu/GII/JP/2010/GII.P2-GII.2/Hiroshima19	LC209473	RdRp
Hu/GII/JP/2010/GII.P2-GII.2/Hiroshima18	LC209472	RdRp
Hu/GII/JP/2009/GII.2/OC09103	AB662872	VP1
Hu/GII/JP/2009/GII.2/OH09034	AB662885	VP1
Hu/GII/JP/2008/GII.2/OH08029–2	AB662869	VP1
Hu/GII/JP/2008/GII.2/OH08009	AB662866	VP1
Hu/GII/JP/2008/GII.2/OC08124	AB662860	VP1
Hu/GII/JP/2007/GII.2/OH07013	AB662865	VP1
Hu/GII/JP/2009/GII.2/OC09044	AB662862	VP1
Hu/GII/JP/2008/GII.P2-GII.2/Hokkaido15	LC209463	RdRp, VP1
Hu/GII/JP/2004/GII.P12-GII.2/Tochigi92	LC209435	RdRp, VP1
Hu/GII/JP/2005/GII.2/OC05041	AB662850	VP1
Hu/GII/JP/2004/GII.2/OC04169	AB279568	VP1
Hu/GII/JP/2002/GII.2/Ina/02	AB195225	VP1
Hu/GII/JP/2015/GII.P2-GII.2/Saitama169	LC209440	RdRp, VP1
Hu/GII/TW/2015/GII.2/15-DS-4	KT962983	VP1
Hu/GII/JP/2013/GII.P16-GII.2/Tochigi46	LC209431	RdRp
Hu/GII/JP/2012/GII.P16-GII.2/Hokkaido18	LC209466	RdRp, VP1
Hu/GII/JP/2014/GII.P16-GII.2/Aomori7	LC145798	RdRp, VP1
Hu/GII/JP/2014/GII.P16-GII.2/Akita8	LC145800	RdRp
Hu/GII/JP/2013/GII.P16-GII.2/Miyagi8	LC209455	VP1
Hu/GII/JP/2014/GII.P16-GII.2/Miyagi2	LC145801	RdRp
Hu/GII/JP/2014/GII.P16-GII.2/Kanagawa52	LC209458	RdRp
Hu/GII/JP/2014/GII.P16-GII.2/Osaka4	LC145802	RdRp, VP1

Strain	GenBank accession no.	Coding region
Hu/GII/JP/2014/GII.P16-GII.2/Saitama126	LC209441	RdRp
Hu/GII/JP/2013/GII.P16-GII.2/Ehime9	LC209475	RdRp
Hu/GII/JP/2013/GII.P16-GII.2/Ehime6	LC209477	RdRp
Hu/GII/JP/2014/GII.P16-GII.2/Ehime4	LC145807	RdRp
Hu/GII/JP/2014/GII.P16-GII.2/Ehime5	LC145808	RdRp
Hu/GII/JP/2012/GII.P16-GII.2/Tochigi30	LC209432	RdRp
Hu/GII/JP/2012/GII.P16-GII.2/Fukui2	LC145790	RdRp
Hu/GII/JP/2012/GII.P16-GII.2/Saitama4	LC145791	RdRp
Hu/GII/JP/2012/GII.P16-GII.2/Ehime2	LC145797	RdRp
Hu/GII/JP/2014/GII.P16-GII.2/Tochigi17	LC209434	RdRp
Hu/GII/JP/2012/GII.P16-GII.2/Saitama5	LC145792	RdRp, VP1
Hu/GII/JP/2012/GII.P16-GII.2/Fukui1	LC145789	RdRp
Hu/GII/JP/2012/GII.P16-GII.2/Osakacity5	LC145793	RdRp
Hu/GII/JP/2012/GII.P16-GII.2/Saitama122	LC209445	RdRp
Hu/GII/JP/2012/GII.P16-GII.2/Tochigi26	LC209433	RdRp
Hu/GII/JP/2012/GII.P16-GII.2/Ehime1	LC145796	RdRp
Hu/GII/JP/2012/GII.P16-GII.2/Hiroshimacity2	LC145794	RdRp
Hu/GII/JP/2014/GII.P16-GII.2/Hiroshimacity6	LC145806	VP1
Hu/GII/JP/2014/GII.P16-GII.2/Hiroshimacity5	LC145805	RdRp
Hu/GII/JP/2012/GII.P16-GII.2/Hiroshimacity1	LC145795	RdRp
Hu/GII/JP/2013/GII.P16-GII.2/Miyagi7	LC209456	RdRp
Hu/GII/JP/2012/GII.P16-GII.2/Akita8	LC145786	RdRp, VP1
Hu/GII/TW/2011/GII.2/CGMH47	KC464505	VP1
Hu/GII/JP/2013/GII.P16-GII.2/Saitama125	LC209442	RdRp
Hu/GII/JP/2014/GII.P16-GII.2/Osaka5	LC145803	RdRp, VP1
Hu/GII/JP/2010/GII.P16-GII.2/Osaka019	LC209454	RdRp
Hu/GII/JP/2009/GII.P16-GII.2/Kanagawa49	LC209461	RdRp
Hu/GII/JP/2009/GII.2/OC09072	AB662870	VP1
Hu/GII/JP/2008/GII.2/OH08020	AB662868	VP1
Hu/GII/JP/2008/GII.2/OC08154	AB662861	VP1
Hu/GII/NL/1999/GII.2/Coevorden191S	AB281081	VP1
Hu/GII/NL/2000/GII.2/DenHaag37	AB281082	VP1
Hu/GII/NL/2000/GII.2/Delft48M	AB281083	VP1
Hu/GII/JP/2002/GII.2/OCS020289	AB279570	VP1
Hu/GII/UK/1989/GII.2/Melksham	X81879	VP1
Hu/GII/U.S./1997/GII.2/Chesterfield/434	AY054300	VP1
Hu/GII/MYS/1978/GII.P2-GII.2/KL109	JX846925	RdRp, VP1
Hu/GII/1976/GII.P2-GII.2/Snow_Mountain	AY134748	RdRp, VP1
Hu/GII/JP/2014/GII.Pe-GII.2/Saitama127	LC209439	RdRp, VP1
Hu/GII/JP/2005/GII.2/OC05010	AB279569	VP1
Hu/GII/U.S./2002/GII.2/TCH-560	KC998960	VP1
Hu/GII/U.S./2002/GII.2/NF2002	JQ320072	VP1
Hu/GII/JP/2002/GII.2/OC02022	AB279556	VP1
Hu/GII/JP/2004/GII.P22-GII.2/OsakaNI	DQ366347	RdRp
Hu/GII/JP/2001/GII.2/OC01243	AB279554	VP1
Hu/GII/RUS/2011/GII.P16/Novosibirsk/Nsk-N1648	KF944111	RdRp

Strain	GenBank accession no.	Coding region
Hu/GII/RUS/2012/GII.P16/Omsk/O1370	KT779557	RdRp
Hu/GII/RUS/2012/GII.P16/Smolensk/S12–31	KF895841	RdRp
Hu/GII/TW/2013/GII.P16/New/Taipei/13-BA-1	KM036380	RdRp
Hu/GII/JP/2016/GII.P16/Kawasaki194	LC175468	RdRp
Hu/GII/JP/2002/GII.P16/Saitama/T87	KJ196286	RdRp
2016 strains		
Hu/GII/JP/2016/GII.P16-GII.2/Kawasaki129	LC215413	RdRp, VP1
Hu/GII/JP/2016/GII.P16-GII.2/Kawasaki151	LC215414	RdRp, VP1
Hu/GII/JP/2016/GII.P16-GII.2/Kawasaki121	LC215415	RdRp, VP1
Hu/GII/JP/2015/GII.P16-GII.2/Ibaraki197	LC213885	RdRp, VP1
Hu/GII/JP/2016/GII.P16-GII.2/Ibaraki253	LC213886	RdRp, VP1
Hu/GII/JP/2016/GII.P16-GII.2/Ibaraki267	LC213887	RdRp, VP1
Hu/GII/JP/2016/GII.P16-GII.2/Ibaraki273	LC213888	RdRp, VP1
Hu/GII/JP/2016/GII.P16-GII.2/Ibaraki290	LC213889	RdRp, VP1
Hu/GII/JP/2016/GII.P16-GII.2/Ibaraki324	LC213890	RdRp, VP1
Hu/GII/JP/2016/GII.P16-GII.2/Ibaraki329	LC213891	RdRp, VP1
Hu/GII/JP/2016/GII.P16-GII.2/Ibaraki374	LC213892	RdRp, VP1
Hu/GII/JP/2016/GII.P16-GII.2/Ibaraki412	LC213893	RdRp, VP1
Hu/GII/JP/2016/GII.P16-GII.2/Ibaraki423	LC213894	RdRp, VP1
Hu/GII/JP/2016/GII.P16-GII.2/Ibaraki472	LC213895	VP1
Hu/GII/JP/2016/GII.P16-GII.2/Ibaraki518	LC213896	RdRp, VP1
Hu/GII/JP/2016/GII.P16-GII.2/Ibaraki536	LC213897	RdRp, VP1
Hu/GII/JP/2016/GII.P16-GII.2/Ibaraki602	LC213898	RdRp, VP1
Hu/GII/JP/2016/GII.P16-GII.2/Ibaraki607	LC213899	RdRp, VP1
Hu/GII/JP/2016/GII.P16-GII.2/Ibaraki636	LC213900	RdRp, VP1
Hu/GII/JP/2016/GII.P16-GII.2/Ibaraki658	LC213901	RdRp, VP1

Reference

1. Kojima S, Kageyama T, Fukushi S, Hoshino FB, Shinohara M, Uchida K, et al. Genogroup-specific PCR primers for detection of Norwalk-like viruses. J Virol Methods. 2002;100:107–14. PubMed http://dx.doi.org/10.1016/S0166-0934(01)00404-9
